# Current Treatment Landscape for Advanced Hepatocellular Carcinoma: Patient Outcomes and the Impact on Quality of Life

**DOI:** 10.3390/cancers11060841

**Published:** 2019-06-18

**Authors:** Daneng Li, Sabrina Sedano, Rebecca Allen, Jun Gong, May Cho, Sunil Sharma

**Affiliations:** 1 Department of Medical Oncology, City of Hope Comprehensive Cancer Center and Beckman Research Institute, Duarte, CA 91010, USA; ssedano@coh.org (S.S.); rallen@coh.org (R.A.); 2 Department of Gastrointestinal Malignancies, Cedars-Sinai Samuel Oschin Comprehensive Cancer Institute, Los Angeles, CA 90048, USA; Jun.Gong@cshs.org; 3 Department of Internal Medicine, UC Davis Comprehensive Cancer Center, Sacramento, CA 95817, USA; maycho@ucdavis.edu; 4 Division of Clinical Sciences, Translational Genomics Research Institute, Phoenix, AZ 85004, USA; ssharma@tgen.org

**Keywords:** hepatocellular carcinoma, systemic therapy, quality of life

## Abstract

Hepatocellular carcinoma (HCC) is the fifth most common cancer and the second leading cause of cancer mortality worldwide. Heterogeneity of clinical conditions contributes to the complex management of care for patients with advanced HCC. Recently, the treatment landscape for advanced HCC has expanded rapidly, with the additional FDA approvals of several oral tyrosine kinase inhibitors (lenvatinib, regorafenib, and cabozantinib), as well as immunotherapies such as immune check point inhibitors (nivolumab and pembrolizumab) and the monoclonal IgG1 antibody, ramucirumab. This expansion has generated a need for novel treatment sequencing strategies in this patient population. In light of these developments, an evaluation of the impact of FDA-approved therapeutics on patient-centered outcomes such as health-related quality of life (HRQoL) is warranted. An increased understanding of HRQoL in patients included in advanced HCC clinical trials could potentially help physician decision-making for treatment sequencing in patients with advanced HCC.

## 1. Introduction

Primary liver cancer is a daunting diagnosis as it is the fifth most common cancer and the second most common cause of cancer mortality worldwide [1]. Hepatocellular carcinoma (HCC) is the main histologic type of liver cancer, accounting for approximately 90% of cases [2,3,4]. Cirrhosis due to chronic hepatitis B virus (HBV) or hepatitis C virus (HCV) is the leading risk factor for the development of HCC, accounting for 80% to 90% of cases [5,6]. HBV is the primary etiological risk factor for HCC on a global scale, particularly in high-rate areas for HCC incidence and developing countries [2,5,6,7]. Additional risk factors for HCC include aflatoxins, excess alcohol consumption, diabetes, nonalcoholic fatty liver disease, and smoking [2].

Although HCC is a leading cause of cancer mortality globally, the disease burden varies by geographical location. HCC distribution is highest in areas with endemic HBV infection, such as Asia and sub-Saharan Africa where incidence rates are greater than 20/100,000 persons [2,3,5,6,7]. Conversely, areas of low-rate for HCC incidence include northern Europe as well as North and South America with incidence rates of less than 10/100,000 persons [2,3,5,6,7]. Although the United States is considered to be a location of low disease burden, the incidence of HCC has nearly tripled since the early 1980s making it the fastest rising cause of cancer-related death [3,8].

Due to the complex nature of the pathogenesis of HCC, treatment decisions depend on a multidisciplinary approach that take into account several factors including size, extent of tumor burden, and functional status of the liver [9]. In general, HCC classification is based on the Barcelona Clinic Liver Cancer (BCLC) staging system, which incorporates information related to liver function, performance status, and tumor burden [7]. The BCLC staging system has 5 defined prognostic subclasses: Stage 0 (very early stage), Stage A (early stage), Stage B (intermediate stage), Stage C (advanced stage), and Stage D and has been linked to a treatment algorithm that allocates specific treatments for each stage [4]. Potential curative therapies include tumor resection, ablation, and liver transplant but are reserved for patients with early stage (BCLC stage 0 or A) HCC [4,10]. Loco-regional therapies such as transarterial chemoembolization (TACE) are often used to control the disease when the neoplasm is confined to the liver (intermediate-stage disease, BCLC stage B) [11,12,13]. However, more than 80% of HCC patients present at advanced stages (BCLC stage C) for which local, curative therapies are not an option and 5-year survival rates are just 18% [11,13,14]. For patients who progress or are not eligible for local treatments, current FDA approved first-line treatments for advanced HCC are the tyrosine kinase inhibitors (TKIs), sorafenib or lenvatinib [15,16,17].

Although the treatment landscape for HCC has recently broadened with the approvals of additional oral TKIs (lenvatinib, regorafenib, and cabozantinib), as well as immunotherapies such as immune checkpoint inhibitors (nivolumab and pembrolizumab) and the monoclonal IgG1 antibody, ramucirumab, the effect of these novel agents on patients’ health-related quality of life (HRQoL) has not been well characterized [15,16,17,18,19,20,21]. Quality of life (QoL) is especially important in this cancer population who have reported feelings of “fear”, “worry”, and “anxiety” upon their diagnosis due to the often poor prognosis associated with advanced HCC [22]. An increased understanding of HRQoL in patients included in advanced HCC clinical trials could provide a foundation to better inform physician decision-making regarding treatment sequencing in this challenging arena.

## 2. Current Treatment Landscape

Before 2007, there was a lack of effective systemic treatment options available for patients with advanced HCC [15,17]. This neoplasm is known to be among one of the most chemoresistant tumors [23]. Consequently, conventional single-agent chemotherapy has not been recommended in HCC due to cytotoxic agents being poorly tolerated by patients with underlying cirrhosis [24]. Overall, combination chemotherapy regimens have also not shown significant promising data, with marginal long-term clinical efficacy [25,26]. As a result, development of systemic therapies for advanced HCC has primarily focused on TKIs and immune checkpoint inhibitors.

### 2.1. Sorafenib

In 2007, the FDA approved sorafenib, an oral TKI targeting–among others–vascular endothelial growth factor (VEGF), the key mediator of angiogenesis, for the first-line treatment of advanced HCC in light of positive results from the Sorafenib Hepatocellular Carcinoma Assessment Randomized Protocol (SHARP) study [15]. This was a multicenter, phase III, double-blind, placebo-controlled trial in treatment-naïve patients with advanced HCC that demonstrated a 2.8-month median overall survival (OS) benefit for sorafenib compared to placebo (10.7 vs. 7.9 months; hazard ratio (HR), 0.69) [15]. A second phase III trial done in the Asia-Pacific region further demonstrated that sorafenib improved median OS compared to placebo (6.5 vs. 4.2 months; HR, 0.68) [17]. Notably, both trials confirmed the antitumor activity of sorafenib in patients with well-preserved liver function (Child–Pugh A) not amenable for surgery or loco-regional therapies [15,17]. The inclusion of patients solely with well-preserved liver function is a common practice in HCC clinical trials used to avoid potential masking of a drug-induced antitumor effect by death from underlying liver disease [27]. Subset analyses of the SHARP trial verified that sorafenib is effective for the treatment of advanced HCC across various etiologies, tumor stages, performance status, and prior treatments [17,28,29].

In addition to single-agent treatment, the benefit of combination therapy with sorafenib has also been investigated in several phase III trials. The results of trials with sorafenib plus erlotinib, doxorubicin, or hepatic arterial infusion chemotherapy (HAIC) failed to improve survival in patients with advanced HCC [30,31,32]. Two phase III trials comparing radioembolization with Y90 to sorafenib were also unsuccessful in improving median OS in patients with locally advanced or recurrent HCC [33,34].

Despite the improvement of overall survival with sorafenib alone, most patients potentially discontinue treatment due to poor tolerance of side effects and dose reductions are common [10,15,35]. The most common adverse events with sorafenib include diarrhea, hand-foot skin reaction, weight-loss, and hypophosphatemia [15,17]. As a result of its marginal efficacy and toxicity profile, other agents have been compared to treatment with sorafenib.

### 2.2. Lenvatinib

In the 10-year period after the FDA approval of sorafenib, several other studies were conducted in search of additional targeted agents to compare to sorafenib. In a randomized, multinational, double-blind, phase III trial comparing brivanib to sorafenib as first-line treatment for HCC showed that brivanib did not improve median OS when compared to sorafenib (9.5 vs. 9.9 months) [36]. Additional head-to-head trials comparing sorafenib with single-agent sunitinib and linifanib were conducted but were also unable to demonstrate superiority or non-inferiority to sorafenib [37,38]. To date, a total of eight first-line trials have failed to meet their primary endpoints versus sorafenib [30,31,32,33,36,37,38,39] (Table 1).

Lenvatinib is an oral TKI with a broad target profile, inhibiting VEGF receptors 1–3, fibroblast growth factor receptors (FGFR) 1–4, platelet-derived growth factor receptor (PDGFR) α, RET, and KIT [40]. The REFLECT trial tested the efficacy and safety of lenvatinib as a first-line treatment for advanced HCC and was the only study that produced statistically significant results during the 10-year period of negative trials [16]. This open-label, multicenter, phase III, non-inferiority trial recruited 954 treatment-naïve patients with advanced HCC. The primary endpoint was met with OS of 13.6 months in the lenvatinib group versus 12.3 months in the sorafenib group (HR: 0.92; 95% CI: 0.79–1.06) showing non-inferiority of lenvatinib with respect to OS compared to sorafenib. In addition, lenvatinib demonstrated statistically significant higher objective response rate (ORR) (24.1% in the lenvatinib arm vs. 9.2% in the sorafenib arm), progression-free survival (PFS) (7.4 vs. 3.7 months), and time-to-progression (TTP) (8.9 vs. 3.7 months) [16].

Although secondary endpoints (ORR, PFS, and TTP) were significantly better with lenvatinib, this agent also had higher rates of grade ≥3 treatment-related treatment-emergent adverse events (57% vs. 49%) [16]. The most frequent adverse events of any grade were arterial hypertension (42%), diarrhea (39%), and decreased appetite (34%). In the sorafenib arm, the most common any grade adverse events were hand-foot skin reaction (52%), diarrhea (46%), and arterial hypertension (30%) [16]. Treatment-related treatment-emergent adverse events led to lenvatinib dose reduction in 37% of patients and drug withdrawal in 9% of patients [16]. The results from the REFLECT trial statistically show that lenvatinib is comparable to sorafenib in terms of OS, and all of the secondary endpoints (ORR, PFS, and TTP) demonstrated statistical and clinical improvements in the lenvatinib group. With these data, lenvatinib is the first TKI since sorafenib approved by the FDA for the first-line treatment of advanced HCC.

### 2.3. Regorafenib

As many studies have demonstrated the heterogeneous nature of HCC, which can lead to primary or acquired resistance to sorafenib [41], the development of second-line therapies for advanced HCC is of interest (Table 2). In the randomized, double-blind, placebo-controlled, phase III RESORCE trial the efficacy and safety of regorafenib in 567 patients with HCC who progressed during sorafenib treatment were evaluated [18]. The primary endpoint was met with median OS of 10.6 months in the regorafenib arm compared to 7.8 months in the placebo arm (HR: 0.63, 95% CI: 0.50–0.79) [18]. The median PFS was 3.1 months with regorafenib versus 1.5 months with placebo (HR: 0.46, 95% CI: 0.37–0.56). The median TTP for regorafenib was 3.2 months compared to 1.5 months with placebo (HR: 0.44, 95% CI: 0.36–0.55).

The patients who were eligible for the RESORCE trial had progressed on sorafenib and were able to tolerate at least 400 mg of sorafenib daily for at least 20 days of the last 28 days of treatment. Similar side effects to that of sorafenib were observed with regorafenib due to similar molecular structures, resulting in more than half of the regorafenib group (51%) requiring dose reductions. Serious adverse events occurred in 44% of patients assigned to regorafenib and 47% of patients assigned to placebo. The most common grade 3 or 4 adverse events for patients treated with regorafenib include hypertension (15%), hand-foot skin reaction (13%), fatigue (9%), and diarrhea (3%) [18]. Based on these data, regorafenib can be selected as a second-line agent for advanced HCC for patients who demonstrated tolerance to sorafenib.

### 2.4. Cabozantinib

In addition to inhibiting angiogenesis by targeting the VEGF signaling pathway, other targets are becoming of interest for the management of advanced HCC. For example, the role of cabozantinib, an oral TKI that targets the mesenchymal-epithelial transition factor (c-Met) pathway as well as the VEGF and RET receptors [42], was analyzed in the randomized, double-blind, phase III CELESTIAL trial [19]. This study compared cabozantinib to placebo in 707 patients who received previous treatment for advanced HCC, had disease progression after at least one systemic treatment, and may have received up to two previous systemic regimens for advanced HCC. This trial showed improved OS in the cabozantinib arm of 10.2 months versus 8.0 months in the placebo arm (HR: 0.76, 95% CI: 0.63–0.92). The secondary endpoints of PFS and ORR were also significantly better in the cabozantinib arm compared to placebo.

Although data from the CELESTIAL trial resulted in notably improved OS, PFS, and ORR for cabozantinib, the rate of adverse events occurring in the cabozantinib arm was high with grade 3 or 4 adverse events in 68% of patients compared to 36% with placebo. The most frequent side effects were hand-foot skin reaction (17%), hypertension (16%), elevated transaminases (12%), fatigue (10%), and diarrhea (10%). The median duration of receiving cabozantinib or placebo was 3.8 months and 2.0 months, respectively, with dose reductions occurring in 62% of the patients in the cabozantinib group and 13% in the placebo group. The rate of discontinuation due to treatment-related adverse events was 16% and 3% in the cabozantinib and placebo arm, respectively [19]. Although this agent is positioned as a broader second- to third-line systemic therapy option, the potential toxicity associated with cabozantinib may require more frequent monitoring of patients with advanced HCC treated with this agent.

### 2.5. Ramucirumab

In an expansion of the treatment of advanced HCC beyond TKIs, ramucirumab, a recombinant immunoglobulin G (IgG) 1 monoclonal antibody, was first investigated in the REACH trial [43]. This study did not meet its primary endpoint of OS, but it did shed light on the effectiveness of this agent on a subgroup of patients who started with ≥400 ng/mL of alpha-fetoprotein (AFP). Consequently, the randomized, double-blind, placebo-controlled, phase III REACH-2 trial was developed [44]. Enrollment was restricted to patients with concentrations of AFP ≥400 ng/mL and who had previously received first-line sorafenib. A total of 292 patients were randomly assigned, 197 to the ramucirumab group and 95 to the placebo group. Median OS significantly improved in the ramucirumab arm compared to placebo (8.5 vs. 7.3 months; HR: 0.71; 95% CI: 0.53–0.95). The median PFS was also significantly improved for ramucirumab at 2.8 months versus 1.6 months with placebo (HR: 0.45; 95% CI: 0.34–0.60). The most common grade 3 or higher treatment-emergent adverse events that occurred in the ramucirumab group were hypertension (13%), hyponatremia (6%), and elevated aspartate aminotransferase (3%). Treatment discontinuation because of any adverse event (18% vs. 11%) or because of treatment-related adverse events (11% vs. 3%) occurred more often in the ramucirumab group than in the placebo group. Based on data from the REACH-2 trial, ramucirumab recently received FDA approval for the treatment of HCC in patients who have AFP of ≥400 ng/mL and previously treated with sorafenib.

### 2.6. Nivolumab

The role of immune checkpoint inhibitors in the treatment of advanced HCC has also been investigated. Nivolumab is a fully human IgG4 monoclonal antibody programmed death 1 (PD-1) inhibitor that leads to the restoration of the antitumor activity of suppressed effector T cells. The safety and efficacy of nivolumab in patients with advanced HCC was assessed in an open-label, non-comparative, dose escalation and expansion, phase I/II trial—CheckMate 040 [20]. In this study, 262 patients were enrolled, irrespective of chronic viral hepatitis etiology, with intermediate or advanced HCC who were intolerant, refused, or progressed on sorafenib. The tumor ORR was 15% and 20% in the dose-escalation and dose-expansion cohort, respectively. In the dose-expansion phase, the 9-month OS rate for nivolumab was 74% (95% CI: 67–79). The value for ORR and the OS rate at 9 months in the second-line treatment of advanced HCC suggests potential benefits of immunotherapy for patients with HCC regardless of the duration of prior sorafenib treatment.

An additional subgroup analysis of CheckMate 040 was conducted for PD-L1 expression levels as a potential biomarker for nivolumab therapy. This analysis showed that patients in the dose expansion phase with PD-L1 ≥ 1% achieved an ORR of 26%, while patients with a PD-L1 < 1% achieved an ORR of 19%, raising the possibility that PD-L1 expression alone might not be a predictive marker for PD-1/PD-L1 inhibitor therapy in patients with HCC. Based on these data, the FDA granted accelerated approval to nivolumab in the second-line setting for advanced HCC and nivolumab is currently being evaluated in a phase III trial against sorafenib as a first-line treatment option (NCT02576509).

### 2.7. Pembrolizumab

Pembrolizumab is another IgG4 anti-PD-1 cancer therapeutic that was tested in the KEYNOTE-224 non-randomized, multicenter, open-label, phase II trial [21]. This study aimed to assess the efficacy and safety of pembrolizumab in advanced HCC patients who had progression on or intolerance to sorafenib. The primary endpoint was ORR and from the 104 patients that were treated, 18 (17%) achieved the ORR and 46 (44%) patients had stable disease. Among the 18 patients who responded to pembrolizumab, there was 1 complete response and 17 partial responses. Grade ≥3 treatment-related adverse events occurred in 26% of the patients; the most common adverse events were elevated levels of aspartate aminotransferase (7%), elevated levels of alanine aminotransferase (4%), and fatigue (4%).

The efficacy data from the KEYNOTE-224 trial led to the accelerated FDA approval of pembrolizumab as a second-line agent for the treatment of patients with advanced HCC who have previously received sorafenib. However, recent results of the confirmatory phase III trial, KEYNOTE-240, revealed that statistically significant improvement of the co-primary endpoints, OS and PFS, was not achieved [45]. Subsequently, many are left with uncertainty regarding the future of single agent PD-1 immune checkpoint inhibitors in the treatment landscape of advanced HCC.

## 3. Quality of Life

Despite recent advances in the treatment landscape for advanced HCC, the overall prognosis for these patients still remains poor, with population-based studies in the United States reporting 1- and 3-year survival rates of merely 20% and 5%, respectively [46]. In light of the poor prognosis associated with advanced HCC, there is a need to prioritize patient centered outcomes such as quality of life (QoL). Particularly, there is a strong evidence base regarding the prognostic value of health-related quality of life (HRQoL), the patients’ individual perception about their life in regards to goals, standards and concerns, and expectations with respect to their diagnosis, in the oncological setting [46,47,48,49,50,51,52]. In spite of this knowledge, there is a paucity of published data evaluating the impact of FDA-approved therapeutics on patient-centered outcomes such as HRQoL for those patients included in advanced HCC clinical trials.

### 3.1. Assessments

The two most commonly used instruments for QoL assessments in advanced HCC clinical trials are the European Organization for Research and Treatment of Cancer (EORTC) QLQ-C30 and the Functional Assessment of Cancer Therapy-Hepatobiliary (FACT-Hep) [53,54]. The EORTC QLQ-C30 is a cancer-specific questionnaire consisting of five functional scales (physical, role, emotional, cognitive, and social), three symptom scales (fatigue, nausea or vomiting, and pain), a global health status and HRQoL scale, and six single items (dyspnea, insomnia, appetite loss, constipation, diarrhea, and financial difficulties) [53]. The EORTC QLQ-HCC18 is an additional 18 question supplement to the EORTC QLQ-C30 designed to assess QoL issues specific to the advanced HCC patient population [48,55]. The EORTC QLQ-HCC18 consists of five multi-item symptom scales (fatigue, jaundice, nutrition, pain, and fever); two single-item symptom scales (abdominal swelling and sexual interest); and one multi-item functional scale (body image) [55]. For both the EORTC QLQ-C30 and HCC18, all scale and item scores are linearly transformed to a scale from 0 to 100. For the functional scales and the global QoL scale of the EORTC QLQ-C30, a high score represents a good level of functioning. Conversely, high scores on the symptom scales and single items of the EORTC QLQ-C30 correspond to more severe symptoms. For all EORTC QLQ-HCC18 scales, a higher score indicates worse symptoms or poorer HRQoL.

The FACT-Hep questionnaire is a 45-item self-report instrument designed to measure HRQoL specifically in patients with hepatobiliary cancer [54]. The FACT-Hep is a combination of the FACT-General (FACT-G) and a specific hepatobiliary module. The original FACT-G is a 27-item compilation that consists of general questions divided into four QoL domains (physical, social/family, emotional, and functional well-being). The additional hepatobiliary module includes 18 specific items relating to hepatobiliary disease symptoms and side effects of treatment. Each item on the FACT-based questionnaires are rated using a five-point Likert scale ranging from 0 (not at all) to 4 (very much). From the FACT-Hep scale, five subscales and an overall HRQoL score are derived with higher scores corresponding to better HRQoL.

### 3.2. Systemic Treatments

In the first global quality of life survey that captured the perspectives of 256 patients diagnosed with HCC, several treatment-related symptoms such as fatigue, sexual dysfunction, abdominal pain, nausea, skin disorders, diarrhea, and alopecia were reported [22]. Of those patients working at the time they started HCC treatment, 60% (*n* = 154) stated the side effects they experienced caused them to stop working [22]. Systemic treatment was reported to negatively affect patients’ relationships with family and caregivers, ability to perform daily activities, and their outlook for the future. Assessing the QoL in patients with advanced HCC plays a crucial role in evaluating the impact of treatment on existing symptoms and the patient’s perspective of their health.

Despite reports demonstrating the effects of treatment on the QoL of HCC patients undergoing curative interventions as well as TACE, there is a dearth of studies that evaluate the effects of first- and second-line systemic therapies on the HRQoL of patients with advanced HCC (Table 3) [56,57,58,59,60,61,62,63,64,65,66,67]. The limited studies that assess the effect of systemic treatment on QoL have been primarily focused on sorafenib as first-line treatment [50,68]. In a QoL study of 36 HCC patients treated with sorafenib using the FACT-Hep questionnaire reported that sorafenib treatment was associated with a drastic decrease in QoL due to adverse events [50]. The adverse events that led to the permanent withdrawal of sorafenib were grade 3 or 4 fatigue, grade 4 thrombocytopenia, grade 3 diarrhea, grade 3 hand-foot skin reaction, and grade 3 hyperbilirubinemia. There were also significant reductions in scores for the FACT-G Physical Well-Being and Functional Well-Being subscales as well as the FACT-Hep total scores 2 months after sorafenib treatment initiation. Interestingly, Shomura et al. found that HRQoL was not significantly impaired in patients who could tolerate sorafenib treatment over the course of 1 year [68]. In a global survey, HCC patients were asked to specify which treatment throughout their treatment journey they found the most challenging excluding surgery and out of 256 patients, 25% thought that sorafenib was the most challenging [22]. Additionally, those patients who most recently had undergone sorafenib therapy reported that it had a negative impact in their QoL [22].

Although there has been a recent expansion of systemic treatment options for HCC, comprehensive QoL data are sparse for these novel therapies. In recently published randomized controlled trials evaluating lenvatinib, regorafenib, cabozantinib, ramucirumab, nivolumab, and pembrolizumab limited QoL data were reported [16,18,19,20,21,43]. The REFLECT trial, which demonstrated the non-inferiority of lenvatinib compared to sorafenib, evaluated QoL using the EORTC QLQ-C30 and EORTC QLQ-HCC18 and found that baseline scores for both questionnaires were similar in the lenvatinib and sorafenib treatment groups. However, upon the initiation of treatment, QoL scores for occupational and social role functioning, pain, diarrhea, body image, and nutrition deteriorated earlier in the sorafenib arm than in the lenvatinib arm [16]. In the RESORCE trial, regorafenib was shown to provide a survival benefit in HCC patients who progressed and were tolerant to sorafenib [18]. Using the FACT-G, FACT-Hep, European Quality of Life-5 Dimensions utility index (EQ-5D-3L) and visual analogue scale (EQ-5D-VAS), HRQoL was assessed as a tertiary outcome and no clinically meaningful differences between the regorafenib and placebo groups were reported [18]. In a sub-analysis of the REACH trial, Chau et al. reported no significant treatment differences in QoL, time to symptomatic deterioration, and performance status deterioration in the intent-to-treat patients with ramucirumab for advanced HCC [69]. Interestingly, in the sub-population of patients with baseline AFP ≥400 ng/mL, ramucirumab significantly improved QoL scores at the end of treatment compared to placebo [69]. Additionally, the post hoc analysis of the CELESTIAL trial revealed that cabozantinib was associated with an initial small reduction in health utility of patients [70]. Nonetheless, upon continued treatment, health utility increased but at the end of the study there was a clinically and statistically significant benefit in mean quality-adjusted life years for cabozantinib [70].

The patient-reported health-status for those being treated with the immune checkpoint inhibitor, nivolumab, as second-line therapy was assessed by utilizing EQ-5D-3L and EQ-5D-VAS in the dose-expansion phase of CheckMate 040 [20]. The data showed that treatment with nivolumab was associated with stable QoL scores with no significant changes in health status and QoL regardless of prior sorafenib treatment [20]. There is an absence of published QoL data for the immune checkpoint inhibitor, pembrolizumab, in the second-line setting for advanced HCC at this time. Ultimately, the need for the evaluation of the detailed impact of these systemic therapies on QoL domains remains a crucial area of research that needs to be addressed in the complex treatment landscape of HCC.

## 4. Current Challenges in the Treatment Landscape

**Figure 1:**
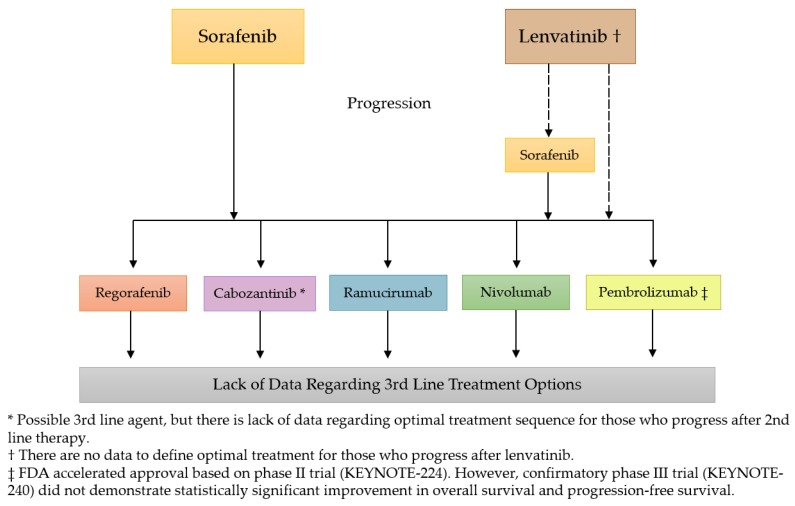
The current treatment landscape for advanced HCC.

Despite recent advances in the development of oral TKIs as well as immune checkpoint inhibitors for the treatment of advanced HCC, physicians are faced with the challenge of sequencing such therapeutic agents (Figure 1). As of today, no additional agent has demonstrated superiority to sorafenib as first-line therapy for advanced HCC. However, the existing caveat for this molecularly targeted agent lies in the fact that a vast majority of patients enrolled into the SHARP and Asia-Pacific studies had well-preserved liver function (Child–Pugh A) and good performance status [15,17]. This is concerning as the majority of HCC patients are diagnosed at advanced stages, generally with chronic cirrhosis, poor liver function, and compromised functional status leaving this subgroup of patients at an increased risk of adverse events and toxicity.

Recently, the FDA approved lenvatinib as a first-line agent for advanced HCC after the REFLECT trial demonstrated the non-inferiority of lenvatinib to sorafenib [16]. Based on the results from the REFLECT trial, lenvatinib seemed to be a promising alternative with better response rates compared to sorafenib and with fewer rates of patients experiencing hand-foot skin reaction (3% vs. 11%) [16]. However, grade 3 or higher treatment-emergent events occurred at similar rates in the lenvatinib and sorafenib arms with more patients experiencing hypertension (23% vs. 14%), decreased appetite (5% vs. 1%), and decreased weight (8% vs. 3%) in the lenvatinib group. Furthermore, patients enrolled in the REFLECT trial could not have >50% liver involvement, clear invasion of the bile duct, or main portal vein invasion suggesting a healthier advanced HCC patient population. Given the differing inclusion criteria between the SHARP and REFLECT trials along with the distinct toxicity profiles of lenvatinib and sorafenib, patient and tumor characteristics should be considered when selecting first-line therapy. The criteria for the differential use of sorafenib or lenvatinib in the first-line setting is a challenge that oncologists face for which selection may depend on outcome priorities, toxicity profiles, and physician comfort with the drugs. While both of these agents had comparable OS, assessing QoL among patients being treated with these novel agents can provide guidance when deciding which treatment is best suited for a specific patient.

Treatment in the second-line setting for advanced HCC initially expanded with the sequential systemic treatment of regorafenib following sorafenib. In a subanalysis of the RESORCE trial, treating patients with advanced HCC with sorafenib followed by regorafenib showed a significant improvement in OS of 26 months from the start of sorafenib treatment compared to 19.2 months in the placebo group [18]. This improved survival time of 26 months rivals the conventional TACE treatment given to patients with intermediate stage HCC [71]. The sequencing of sorafenib-regorafenib may provide those with advanced HCC a similar prognosis to those diagnosed with intermediate stage HCC. However, it is important to note that this positive outcome is only possible for a subgroup of patients with advanced HCC who were able to both tolerate and had tumor progression on sorafenib as first-line therapy. In addition to regorafenib, cabozantinib was also added to the second-line setting for the treatment of advanced HCC following the positive results from the CELESTIAL trial [19]. Notably, the CELESTIAL trial allowed the inclusion of patients tolerant and intolerant to sorafenib, whereas the phase III trial with regorafenib only included the sub-group of patients who were tolerant to sorafenib. Additionally, the CELESTIAL trial had broad inclusion criteria allowing for patients who received up to two prior lines of systemic treatment. However, data supporting use of cabozantinib in the third-line setting remains limited due to the small number of patients having received two prior lines of systemic therapy enrolled on CELESTIAL.

In addition to TKIs, the treatment landscape of advanced HCC has also incorporated the use of a monoclonal antibody (IgG1), ramucirumab, and immune checkpoint inhibitors, pembrolizumab and nivolumab, in the second-line setting. These novel agents may serve as second-line systemic therapy options in those with poor tolerance to the oral TKIs discussed above. The REACH-2 trial data suggests that ramucirumab is an option for the cohort of patients with elevated baseline AFP levels (≥400 ng/mL) given the statistically significant 1.2 months improvement of median OS compared to placebo [44]. However, discussions have risen in regards to AFP being considered a predictive biomarker when assessing the treatment of patients with advanced HCC [72,73]. When analyzing across all HCC clinical trials, it is important to note that varying values were used to categorize high or low AFP concentrations. The KEYNOTE 224 trial and REFLECT trial both used 200 ng/mL as the concentration cutoff to differentiate between high and low AFP levels while the CELESTIAL and RESORCE trials used 400 ng/mL as the stratification measure [16,18,19,21]. Interestingly, the phase II/III trials aforementioned showed response in advanced HCC patients regardless of initial AFP levels [16,18,19,21]. This brings to question whether baseline AFP level can be used as a true predictive biomarker of response to various systemic therapies for advanced HCC. Although ramucirumab provides an alternative option to advanced HCC patients who progress on sorafenib, further research needs to be conducted to better define where ramucirumab truly fits into the paradigm of treatment sequencing for advanced HCC.

The role of immune checkpoint inhibitors has further complicated the treatment landscape for advanced HCC. Nivolumab, received accelerated approval from the FDA in the second-line setting due to the promising results from CheckMate 040 and is currently undergoing a phase III head-to-head trial against sorafenib as a first-line therapeutic intervention for advanced HCC (CheckMate 459) [20]. If statistically significant data result from CheckMate 459, the addition of nivolumab in the first-line setting will redefine the landscape of the existing first- and second-line systemic treatments for advanced HCC. Further research will be needed to rearrange and reassess the roles of the current second-line therapies with nivolumab as the first-line agent. Furthermore, in light of the failure of the confirmatory trial for pembrolizumab to meet its primary endpoints [45,74], the true benefit derived from single-agent PD-1 immune checkpoint inhibitors for patients with advanced HCC is unclear. Therefore, the pending results of the CheckMate 459 trial will likely serve to further illuminate the true role of PD-1 immune checkpoint inhibitors in the advanced HCC treatment landscape.

It is worth noting that all these second-line therapies have only been investigated among patients who had prior sorafenib exposure. Currently, there is a lack of data regarding the extrapolation of the results of these trials in patients who have been previously treated with lenvatinib as first-line therapy for advanced HCC. Therefore, studies focused on the efficacy and safety for the use of second-line agents (regorafenib, cabozantinib, ramucirumab, nivolumab, and pembrolizumab) among patients who only had prior systemic treatment with lenvatinib are needed.

With the expanding armamentarium for treatment of advanced HCC, sequencing is further complicated by the similar efficacies and tolerability between agents in the same line of treatment. Thus, there is a need to consider additional factors beyond traditional primary endpoints of survival and time to progression when determining the sequence of treatment. QoL is one such factor that may assist the complexity of decision making and will bring the patient voice into clinical setting. While there is a foundation for the prognostic value of QoL in the oncological setting [46,47,48,49,50,51,52], it was previously recommended that QoL assessment only serve as ancillary information in clinical trials, rather than a primary endpoint, due to the lack of validated assessments [27]. However, since the release of these guidelines for HCC clinical trials, QoL assessments, including the EORTC QLQ-C30 and FACT-Hep have been validated for those with advanced HCC and are recommended for use in research studies [55,75,76]. Therefore, obtaining additional QoL data in conjunction with traditional efficacy and safety analyses for the current first- and second-line systemic therapy agents in advanced HCC is warranted.

## 5. Current and Future Research

Advanced HCC treatment is rapidly evolving as several phase III clinical trials are currently under investigation. Two trials are investigating the first-line use of single agent immunotherapies. For example, nivolumab is being evaluated in a head-to-head phase III clinical trial, CheckMate-459, against sorafenib as a first-line treatment in patients with advanced HCC (NCT02576509) [77]. In addition, Icaritin, an oral immunotherapy agent, underwent evaluation for treating advanced HCC in a phase Ib trial and was found to be tolerable among patients with no grade 3 or above adverse events [78]. Because of the promising antitumor activities reported, Icaritin is currently being tested in a phase III clinical trial against sorafenib in the first-line setting for patients with advanced HCC (NCT03236649).

Additionally, there are several active studies examining the combination of targeted therapies with immune checkpoint inhibitors. Bevacizumab, a monoclonal antibody, is being evaluated in combination with atezolizumab, a PD-L1 inhibitor, compared to sorafenib in the open-label, randomized, phase III IMbrave 150 trial (NCT03434379) [79]. Participants with locally advanced or metastatic HCC who have received no prior systemic treatment will be accrued. Atezolizumab is also being evaluated in combination with cabozantinib against sorafenib in patients with previously untreated advanced HCC in COSMIC-312 (NCT03755791). Furthermore, due to promising data from an open-label phase Ib trial, the safety and efficacy of lenvatinib in combination with pembrolizumab versus lenvatinib is also being evaluated in a phase III clinical trial as first-line therapy for patients with advanced HCC (NCT03713593).

Other innovative approaches have emerged in advanced HCC clinical trials such as the combination of Yttrium-90 (Y90) transarterial radioembolization with immunotherapy. Radiation has been found to enhance the antitumoral immune response of immune checkpoint inhibitors and is considered an inducer of immunogenic cell death leading to improved immunogenicity [80]. Based on these findings, an Asian phase II open-label, non-randomized trial of Y90 in combination with nivolumab is currently recruiting patients with advanced HCC (NCT03033446) [81]. This combination is also being evaluated in the United States in a phase I/Ib trial that is currently recruiting advanced HCC patients (NCT02837029).

The role of dual immune checkpoint inhibitors is also being investigated in the up-front treatment setting for advanced HCC. Durvalumab, a PD-L1 inhibitor, plus tremelimumab, a fully human IgG2 monoclonal antibody directed against human cytotoxic T lymphocyte-associated antigen 4 (CTLA4) combination therapy versus durvalumab monotherapy versus sorafenib is being evaluated in the HIMALAYA trial (NCT03298451) [82]. The advent of all these new HCC clinical trials incorporating the combination of targeted therapies with immune checkpoint inhibitors as well as single agent or combination immunotherapy approaches may result in the expansion of the HCC armamentarium giving advanced HCC patients and physicians a wider array of treatment options. With this expansion, HRQoL measures will be even more crucial in aiding both patients and physicians in deciding the ideal sequence and selection of treatment for advanced HCC patients.

## 6. Conclusions

Hepatocellular carcinoma is one of the most malignant neoplasms worldwide, with a majority of cases presenting at advanced stages. Irrespective of the recent expansion in treatment options for patients diagnosed with advanced HCC, the prognosis of this increasing patient population still remains poor. The development of concrete sequencing strategies with both a patient’s QoL and survival as primary endpoints is an unmet need that this cancer population faces when undergoing therapeutic interventions. Since the approval of sorafenib in the first-line setting for advanced HCC in 2007, there has been a recent explosion of additional agents that had positive results in large phase III clinical trials. The non-inferiority of lenvatinib in the first-line setting and the addition of regorafenib, cabozantinib, ramucirumab, nivolumab, and pembrolizumab as second-line therapeutic agents could contribute to improved outcomes for HCC patients. Nevertheless, the incorporation of oral TKIs and immune checkpoint inhibitors as therapeutic interventions has brought upon a challenge to physicians regarding the optimal sequencing of these novel treatments. The positive trials that led to the approval of the current second-line oral TKIs and immune checkpoint inhibitors had differing eligibility criteria, leaving to the question of whether those patients that were not included could find benefit in being treated with such agents. In addition, the side effect profiles and tolerability to treatment of the various first- and second-line systemic therapies is a major concern in conserving QoL of HCC patients with reduced life expectancy. The limited data assessing the effects of systemic treatment on QoL in this patient population is a gap in knowledge that must be addressed in order to bring the patient voice into clinical settings. An increased understanding of the HRQoL of patients included in current and future HCC clinical trials may provide a foundation to inform physician decision-making for treatment sequencing in patients with advanced HCC.

## Figures and Tables

**Table 1:** Clinical trials for first-line treatment of advanced HCC.

Study Name	Design	Met Primary Endpoint(s)	Patients (*n*)	TTP/PFS (Months)	mOS (Months)
**1st Line Setting**					
SHARP	Sorafenib vs. placebo	Yes	Sorafenib (299) Placebo (303)	5.5 vs. 2.8 HR = 0.58 95% CI: 0.45–0.74 *p* < 0.001	10.7 vs. 7.9 HR = 0.69 95% CI: 0.55–0.87 *p* < 0.001
Asian-Pacific	Sorafenib vs. placebo	Yes	Sorafenib (150) Placebo (76)	2.8 vs. 1.4 HR = 0.57 95% CI: 0.42–0.79 *p* < 0.001	6.5 vs. 4.2 HR = 0.68 95% CI: 0.50–0.93 *p* = 0.014
SUN1170	Sunitinib vs. sorafenib	No	Sunitinib (530) Sorafenib (544)	4.1 vs. 3.8 HR = 1.13 95% CI: 0.98–1.31 *p* = 0.308	7.9 vs. 10.2 HR = 1.30 95% CI: 1.13–1.50 *p* = 0.001
BRISK-FL	Brivanib vs. sorafenib	No	Brivanib (577) Sorafenib (578)	4.2 vs. 4.1 HR = 1.01 95% CI: 0.88–1.16 *p* = 0.853	9.5 vs. 9.9 HR = 1.06 95% CI: 0.93–1.22 *p* = 0.373
LIGHT	Linifanib vs. sorafenib	No	Linifanib (514) Sorafenib (521)	5.4 vs. 4.0 HR = 0.76 95% CI: 0.64–0.90 *p* = 0.001	9.1 vs. 9.8 HR = 1.04 95% CI: 0.90–1.22 *p* = NS
SEARCH	Sorafenib +/− erlotinib	No	Sorafenib + Erlotinib (362) Sorafenib + placebo (358)	3.2 vs. 4.0 HR = 1.135 95% CI: 0.94–1.37 *p* = 0.180	9.5 vs. 8.5 HR = 0.929 95% CI: 0.78–1.11 *p* = 0.408
CALGB80802	Sorafenib +/− doxorubicin	No	Sorafenib + Doxorubicin (180) Sorafenib + Placebo (176)	3.6 vs. 3.2 * HR = 0.90 95% CI: 0.72–1.20 *p* = NS	9.3 vs. 10.5 HR = 1.06 95% CI: 0.80–1.40 *p* = NS
REFLECT	Lenvatinib vs. sorafenib	Yes	Lenvatinib (478) Sorafenib (476)	8.9 vs. 3.7 HR = 0.63 95% CI: 0.53–0.73 *p* < 0.0001	13.6 vs. 12.3 HR = 0.92 95% CI: 0.79–1.06 *p* = NS
SARAH	Y90 vs. sorafenib	No	Y90 (237) Sorafenib (222)	4.1 vs. 3.7 * HR = 1.03 95% CI: 0.85–1.25 *p* = 0.760	8.0 vs. 9.9 HR = 1.15 95% CI: 0.94–1.14 *p* = 0.180
SIRveNIB	Y90 vs. sorafenib	No	Y90 (182) Sorafenib (178)	6.1 vs. 5.4 HR = 0.88 95% CI: 0.70–1.10 *p* = 0.290	8.8 vs. 10.0 HR = 1.10 95% CI: 0.90–1.40 *p* = 0.360

* Progression-Free Survival (PFS); NS: not significant.

**Table 2:** Clinical trials for second-line treatment of advanced HCC.

Study Name	Design	Met Primary Endpoint(s)	Patients (*n*)	TTP/PFS (months)	mOS (months)
**2nd Line Setting**					
RESORCE	Regorafenib vs. placebo	Yes	Regorafenib (379) Placebo (194)	3.2 vs. 1.5 HR = 0.44 95% CI: 0.36–0.55 *p* < 0.0001	10.6 vs. 7.8 HR = 0.63 95% CI: 0.50–0.79 *p* < 0.0001
CELESTIAL	Cabozantinib vs. placebo	Yes	Cabozantinib (470) Placebo (237)	5.2 vs. 1.9 * HR = 0.44 95% CI: 0.36–0.52 *p* < 0.001	10.2 vs. 8.0 HR = 0.76 95% CI: 0.63–0.92 *p* = 0.005
REACH-2	Ramucirumab vs. placebo	Yes	Ramucirumab (197) Placebo (95)	3.0 vs. 1.6 HR = 0.43 95% CI: 0.31–0.58 *p* < 0.0001	8.5 vs. 7.3 HR = 0.71 95% CI: 0.53–0.94 *p* = 0.020
CheckMate 040	Nivolumab phase I/II	Yes	Dose-escalation (48) Dose-expansion (214)	Dose-escalation: 3.4 Dose-expansion: 4.1	Dose-escalation: 15.0 Dose-expansion: NR
KEYNOTE-224	Pembrolizumab phase II	Yes	Pembrolizumab (104)	4.9 95% CI: 3.9–8.0	12.9 95% CI: 9.7–15.5
KEYNOTE-240	Pembrolizumab vs. placebo	No	Not provided	Not provided HR = 0.78 95% CI: 0.61–0.99 *p* = 0.021	Not provided HR = 0.78 95% CI: 0.61–1.00 *p* = 0.024

* Progression-Free Survival (PFS); NR: not reached.

**Table 3:** Health-Related Quality of Life of systemic treatments.

Agent	Study Type	No. of Patients	HRQoL Assessment Tool	Scale(s)/Domain(s)	Outcome
Sorafenib	Prospective	36	FACT-Hep	Physical well-being	Score decrease was detected from baseline to week 1, with a median reduction of −8.3 (range: −60.1 to −17.9; *p* = 0.0003)
Sorafenib	Prospective	54	SF-36 (Japanese Version)	All domains	Scores >40 maintained over a 1-year period (*n* = 13)
				Physical functioning	Baseline scores ≥40 significantly associated with longer overall survival (*p* = 0.053)
				Social functioning	Baseline scores ≥40 associated with longer treatment duration (*p* = 0.016)
Lenvatinib	Phase III Trial	954	EORTC QLQ-C30, EORTC QLQ-HCC18	EORTC QLQ-C30: role functioning, pain, and diarrhea EORTC QLQ-HCC18: nutrition and body image	Clinically meaningful deterioration observed later in lenvatinib compared to sorafenib
				EORTC QLQ-C30 summary score	No significant difference between lenvatinib and sorafenib (HR = 0.87, 95% CI: 0.75–1.01)
Regorafenib	Phase III Trial	573	FACT-G, HACT-Hep, EQ-5D, EQ-VAS	All scales and domains	No clinically meaningful differences between regorafenib and placebo
Ramucirumab	Subanalysis	565	FACT Hepatobiliary Symptom Index (FHSI)-8, EuroQoL (EQ-5D)	All scales and domains	No significant treatment differences
				FHSI-8	Subpopulation of patients with baseline AFP ≥400ng/mL had significantly reduced deterioration at the end of treatment compared with placebo (*p* = 0.038)
Cabozantinib	Subanalysis	707	EQ-5D-5L	Quality-adjusted life years (QALY)	Mean accrued QALYs with cabozantinib was +0.115 vs. placebo (95% CI: 0.032 to 0.198; *p* = 0.007)
Nivolumab	Phase I/II Trial	262	EQ-5D-3L, EQ-5D-VAS	All scales and domains	Stable patient-reported outcomes despite previous treatment with sorafenib
